# The Oncolytic Virus *dl*922-947 Triggers Immunogenic Cell Death in Mesothelioma and Reduces Xenograft Growth

**DOI:** 10.3389/fonc.2019.00564

**Published:** 2019-07-12

**Authors:** Sarah Di Somma, Carmelina Antonella Iannuzzi, Carmela Passaro, Iris Maria Forte, Raffaella Iannone, Vincenzo Gigantino, Paola Indovina, Gerardo Botti, Antonio Giordano, Pietro Formisano, Giuseppe Portella, Anna Maria Malfitano, Francesca Pentimalli

**Affiliations:** ^1^Dipartimento Scienze Mediche Traslazionali, Università di Napoli “Federico II”, Naples, Italy; ^2^Cell Biology and Biotherapy Unit, Istituto Nazionale Tumori IRCCS, Fondazione G. Pascale, Naples, Italy; ^3^Pathology Unit, Istituto Nazionale Tumori IRCCS, Fondazione G. Pascale, Naples, Italy; ^4^Center for Biotechnology, Sbarro Institute for Cancer Research and Molecular Medicine, College of Science and Technology, Temple University, Philadelphia, PA, United States; ^5^Scientific Direction, Istituto Nazionale Tumori IRCCS, Fondazione G. Pascale, Naples, Italy; ^6^Department of Medical Biotechnologies, University of Siena, Siena, Italy

**Keywords:** virotherapy, oncolytic virus, *dl*922-947, mesothelioma, immunogenic cell death

## Abstract

**Background:** Malignant pleural mesothelioma (MPM) is an aggressive cancer associated with asbestos exposure that urgently requires effective therapeutic strategies. Current treatments are unable to increase significantly patient survival, which is often limited to <1 year from diagnosis. Virotherapy, based on the use of oncolytic viruses that exert anti-cancer effects by direct cell lysis and through the induction of anti-tumor immune response, represents an alternative therapeutic option for rare tumors with limited life expectancy. In this study, we propose the use of the adenovirus *dl*922-947, engineered to allow selective replication in cancer cells, to counteract MPM.

**Methods:** We performed a thorough preclinical assessment of *dl*922-947 effects in a set of MPM cell lines and xenografts. Cytotoxicity of *dl*922-947 alone and in combination assays was evaluated by sulforhodamine B assay. Cell cycle, calreticulin expression, and high mobility group box protein 1 (HMGB1) secretion were determined by flow cytometry, whereas ATP content was determined by a luminescence-based bioassay. The modulation of angiogenic factors in MPM-infected cells was evaluated through ELISA.

**Results:** We found that *dl*922-947 infection exhibits cytotoxic effects in MPM cell lines, affecting cell viability, cell cycle progression, and regulating main hallmarks of immunogenic cell death inducing calreticulin surface exposure, HMGB1 and ATP release. Our results also suggest that *dl*922-947 may affect angiogenic signals by regulation of VEGF-A and IL-8 secretion. Furthermore, *dl*922-947 shows anti-tumor efficacy in murine xenograft models reducing tumor growth and enhancing survival. Finally, the combination with cisplatin potentiated the cytotoxic effect of *dl*922-947.

**Conclusions:** Overall our data identify virotherapy, based on the use of *dl*922-947, as a new possible therapeutic strategy against MPM, which could be used alone, in combination with standard chemotherapy drugs, as shown here, or other approaches also aimed at enhancing the antitumoral immune response elicited by the virus.

## Introduction

Malignant mesothelioma (MM) is an aggressive tumor type with very poor prognosis. MM is considered a rare cancer, nonetheless it is estimated that 30,443 new cases will be diagnosed and 25,576 deaths will occur worldwide in 2018 only, therefore contributing importantly to the global cancer burden (1). The main etiologic factor for MM development is exposure to asbestos, a term that includes six types of carcinogenic mineral fibers that are used commercially (2). Indeed, incidence and mortality rates are highly variable in areas characterized by the industrial use of asbestos, such as naval shipyards, asbestos–cement plants and others (3). Although asbestos use has been banned in many countries, some forms are still mined and used worldwide and other mineral fibers, similar to asbestos, are not even regulated, so there is increasing concern for the development of more MM cases in the future (2). Also, the risk associated with environmental exposure is perceived as increased (4). An additional issue of concern is that MM is characterized by long latency, of usually three or four decades from first asbestos exposure, therefore, despite the ban, a high number of cases is still expected in various countries (5). MM affects mostly the pleura (93.9%), but also the peritoneum (5.8%), the tunica vaginalis (0.3%), and the pericardium (0.1%) (6). According to the most recent World Health Organization (WHO) classification, malignant pleural mesotheliomas (MPM) can be divided into three main subtypes: epithelioid (which is the most frequent type), sarcomatoid and biphasic (7). MPM is resistant to currently available therapies and even with multimodal treatment including surgery, radiation, and/or chemotherapy, the prognosis remains dismal with a median survival of approximately 16–29 months depending on tumor stage and histotype (8). At present, most attempted targeted approaches against MPM, aimed at counteracting a wide range of cancer hallmarks, have failed in the clinical setting, prompting the identification of innovative therapeutic strategies (9). Cancer therapy through oncolytic viruses (OVs) has always been considered a promising therapeutic approach and virotherapy-based strategies have recently found a successful application in the clinical setting (10, 11).

MM represents an ideal candidate for virotherapy for numerous reasons including the frequently localized pattern of growth and the pleural location, which allows direct access for the intra-tumoral injection of the OVs. (12). OVs selectively replicate in and kill cancer cells (10), with a direct lytic effect. Beyond the lytic effects, OVs have indirect effects, such as the induction of a robust anti-tumoral immune response, either innate or adaptive (13), and the re-shaping of tumor microenvironment (TME) (14). In particular, OV infection leads to the release of cytokines, tumor-associated antigens (TAAs), and danger signals, such as damage-associated molecular pattern molecules (DAMPs) and pathogen-associated molecular pattern (PAMPs) molecules, activating immunogenic cell death (ICD) (15, 16) and stimulating an immune response against cancer cells (17, 18). Dying cells release a variety of cytokines such as interferons (IFNs), tumor necrosis factor (TNF)-alpha and interleukins, which promote the immune response and modulate TME toward an anti-tumoral phenotype (19, 20).

Among OVs, adenoviruses (Ads) are particularly attractive. Our group has extensively analyzed the anti-tumor effects of *dl*922-947, an adenoviral mutant bearing a 24 bp deletion in the E1A-Conserved Region 2 (21–23). By deleting this domain, viral replication can only proceed in cells with a defective retinoblastoma (RB) pathway, an almost universal abnormality in human malignancies, including MM in which mutations affecting this pathway, such as deletion of the *CDKN2A* locus encoding the RB upstream regulator p16, is among the most common (24).

We and others showed that *dl*922-947 is effective against cancer cells of different origin both alone or in combination with other therapeutic agents (25–28). *dl*922-947 also exerts an anti-angiogenic effect and mediates the reduction of tumor associated macrophages (TAMs) (29). However, the responsee to OVs is cell-type dependent, making it necessary to assess the effects of a specific virus in the context of each cancer cell type.

For all the above considerations and given that MPM is characterized by a pro-tumor microenvironment, we set out to assess the efficacy of *dl*922-947 against MPM, its ability to modulate the production of proangiogenic cytokines and its effects in combination assays.

## Materials and Methods

### Cells and Adenoviruses

The NCI-H28, NCI-H2452, MSTO-211H, and NCI-H2052 MPM cell lines were purchased from American Type Culture Collection (ATCC). Cells were cultured in RPMI-1640 supplemented with 10% fetal bovine serum (ThermoFisher) in standard conditions (5% CO_2_ at 37°C). Cells undergoing exponential growth were used for all the experiments. *dl*922-947 viral stocks were expanded in the human embryonic kidney cell line HEK-293, purified, stored and quantified (1.22 × 10^8^ p.f.u./ml) as previously described (30).

### Cell Viability Assay

NCI-H28, NCI-H2452, MSTO-211H, and NCI-H2052 cells were seeded in triplicates in 96-well plates at a density of 500 cells/well for MSTO-211H and 800 cells/well for the other cell lines and allowed to adhere for 24 h. Cells were then infected with *dl*922-947 at doses ranging from 0.34 to 250 p.f.u/cell for 5 days. At the end of the treatment cells were fixed with 50% v/v trichloroacetic acid and stained with 0.4% w/v sulforhodamine B (SRB) in 1% v/v acetic acid as previously described (28). The percentage of cell viability after treatment was calculated assuming as 100% the number of untreated cells. The concentration of *dl*922-947 or cisplatin required to inhibit 50% of cell viability (half maximal inhibitory concentration, IC50) was determined by a dose-response curve using GraphPad Prism 7 Software.

### *In vitro* Evaluation of *dl*922-947 DNA Amplification

MSTO-211H (10 × 10^4^ cells × well) and NCI-H28 cells (5 × 10^4^ cells × well) were seeded in duplicate in 12-well-plates and 24 h later infected with viruses at the IC50 and with AdGFP. Cells and supernatants were separately collected 24 and 48 h post-infection. Cell pellets were disrupted by three freeze–thaw cycles to release the virus, then were centrifuged at 1,000 g for 5 min and supernatants were collected. Viral DNA was extracted by High Pure Viral Nucleic Acid Kit (Roche) and quantified by Real-Time PCR using the primers for viral HEXON gene expression in both supernatants (an indirect measure of released viral particle) and cellular pellets (intracellular virus) as previously reported (28). From purified DNA and using a UV spectrophotometer, we obtained 1.42 × 10^12^ viral particles (vp)/ml.

### Cell Cycle Progression Assay

MSTO-211H (2.5 × 10^5^ cells) and NCI-H28 cells (5 × 10^5^ cells) were seeded in 100 mm plates and after 24 h infected with *dl*922-947 or with a control, the adenovirus wild type (Adwt) used at the same pfu of *dl*922-947. Then, a kinetic was performed harvesting cells at 24, 48, and 72 h post-infection (hpi) to determine the effects of viral infection on cell cycle progression. After the incubation, total cell populations were fixed in ice-cold 70% ethanol and then stained with a 40 μg/ml propidium iodide (PI) solution containing 20 μg/ml RNase A. After 20 min incubation at room temperature, samples were analyzed by flow cytometry. All samples were acquired at BD LSRFortessa (BD Biosciences, San Jose, CA, USA) and analyzed by BD FACSDiva Software.

### Intracellular ATP Content

MSTO-211H and NCI-H28 cells (2 × 10^3^ cells/well) were cultured in triplicates in 96-well plates. After 24 h cells were infected with *dl*922-947 used at its corresponding IC50. As a positive control, we used the histone deacetylase (HDAC) inhibitor SAHA (5 μg/ml), which is known to trigger cell death in MPM cells (31). Forty-eight hpi cells were processed according to the manufacturer's instructions of the ATPlite luminescence detection kit (Perkin Elmer).

### Calreticulin Surface Expression Marker

MSTO-211H (1 × 10^4^) and NCI-H28 (1 × 10^4^) cells were seeded in 24-well-plates and after 24 h infected with *dl*922-947 used at its corresponding IC50 or with SAHA (5 μg/ml). 48 hpi and 72 hpi cells were harvested, washed with PBS 1X and stained with anti-calreticulin-PE conjugated antibody (Enzo Life Sciences) for 15' on ice at room temperature. Cells were then washed with PBS 1X, acquired through the BD LSRFortessa flow cytometer (BD Biosciences, San Jose, CA, USA) and analyzed by BD FACSDiva Software.

### High Mobility Group Box Protein 1 (HMGB1) Intracellular Staining

MSTO-211H (1.5 × 10^3^) and NCI-H28 (5 × 10^3^) cells were seeded in 96-well-plates. After 24 h cells were infected with *dl*922-947 used at its corresponding IC50 or with SAHA (5 μg/ml). Cells were harvested at 48 and 72 hpi and 4 h before the end of the treatment, brefeldin A (10 μg/ml) (Sigma) was added to the cultures to block the transport to the extracellular space. Upon collection, cells were washed with PBS 1X, fixed in 4% paraformaldehyde for 10 min at room temperature and then, after 1 min on ice, permeabilized with 90% methanol added drop by drop on vortex. After further 10 min on ice, cells were kept at −20°C overnight before HMGB1 intracellular staining. Cells were washed with PBS 1X, re-suspended in incubation buffer (0.2% BSA, 0.05% NaN_3_ in PBS 1X) and stained with anti-HMGB1-PE conjugated antibody (BioLegend) for flow cytometry analyses.

### ELISA Assay

To assess IL-8 and VEGF-A production MSTO-211H (2.5 × 10^4^) and NCI-H28 (2.5 × 10^4^) cells were seeded in 12-well-plates and, after 24 h, infected with *dl*922-947. Cell supernatants were isolated at 48 hpi to perform ELISA assays according to manufacturer's instructions of Human IL-8 (ELISA, Thermi Fisher) and Human VEGF (DuoSet ELISA, RD system) kits.

### *In vivo* Assay

All experiments were performed in 6-week-old female CD1 athymic mice (Charles-River, Italy). MM xenografts were obtained as previously described (32). Briefly, MSTO-211H (2 × 10^6^) cells were injected into the right flank of 18 athymic mice. After 40 days, tumor volume was evaluated and the animals were divided into two groups (9 animals/group) with similar average xenograft size. Mice received an intra-tumor injection of *dl*922-947 (4 × 10^6^ p.f.u.) twice per week for 5 weeks. Tumor diameters were measured with calipers and tumor volumes (V) were calculated by the rotational ellipsoid formula: V = A × B^2^/2 (A = axial diameter, B = rotational diameter). Mice were maintained at the Animal Facility of the Department of Scienze Mediche Traslazionali. All animal experiments were conducted in compliance with current Italian regulations for the welfare of animals used in studies of experimental neoplasia.

### Tissue Histology

Tumor samples were aseptically excised with a part of surrounding normal tissues, fixed in 10% neutral buffered formalin and embedded in paraffin before sectioning and staining. Serial sections (4 μm) were obtained and subsequently deparaffinized in xylene and rehydrated in an ethanol series. Hematoxylin and eosin (H & E) staining was performed according to standard protocols for histopathology evaluation. The slides were examined by two pathologists using an Olympus BX43® light microscope (Olympus, Tokyo, Japan) and images were photographed and acquired by digital camera DP21. Immunohistochemical expression of CD31 was performed on 4 μm FFPE tissue slides in accordance with previous work (27). The positive cells were evaluated by examining at least four fields at 200X magnification.

### Drug Combination Studies

For drug combination studies, we first determined, through SRB assay (as described above), the IC50 values at 96 h of cisplatin (Calbiochem) in NCI-H28 and MSTO-211H. Cisplatin was dissolved in DMSO (Sigma Aldrich) to achieve a 20 mM stock solution. Subsequently, based on the IC50 values, serial dilutions of *dl*922-947, and cisplatin were combined in various doses at a constant ratio. Synergism, additivity, or antagonism were determined upon calculation of the combination index (CI) through the CompuSyn software 1.0 (ComboSyn, Inc. Paramus, NJ. 07652 USA) based on the Chou-Talalay equation. CI <1 indicates synergism, CI = 1 additive effect, and CI >1 antagonism. The reported r value represents the linear correlation coefficient of the median-effect plot and indicates the data conformity to the mass-action law.

### Statistical Analyses

Statistical analyses were performed by Prism 7 (GraphPad software), and *p*-values were calculated as indicated in the figure legends.

## Results

### *dl*922-947 Infection Induces Cytotoxic Effects in MPM Cell Lines

**Figure 1:**
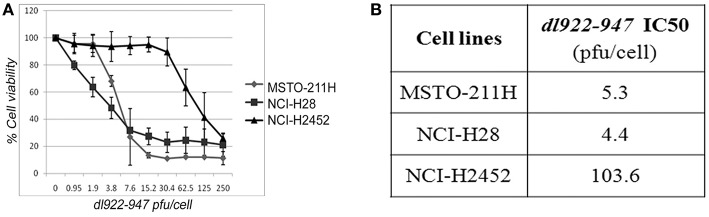
Cytotoxic effect of *dl*922-947 in MPM cell lines. **(A)** Dose-response curves obtained through SRB assay assessing cell viability in MPM cell lines 5 days after treatment with *dl*922-947 at the indicated concentrations. The results are reported as the means ± s.d. of at least 2 independent experiments, each conducted in triplicate, and expressed as percentages of cell viability calculated with respect to the control cells treated with DMSO alone. **(B)** Table reporting the *dl*922-947 IC50 values, determined through SRB assay upon 5 days of treatment as calculated by GraphPad Prism 7.

We first assessed, through SRB assay, the effect of *dl*922-947 on a panel of cell lines (NCI-H28, NCI-H2452, MSTO-211H, and NCI-H2052) derived from human MPMs of the main different histotypes—epithelioid, biphasic and sarcomatoid, respectively. We observed that *dl922-*947 infection induced a dose-response cytotoxic effect in MSTO-211H, NCI-H28, and NCI-H2452 cell lines showing an IC50 of 5.3, 4.3, and 103.6 p.f.u./cell, respectively (Figure 1). *dl*922-947 infection did not affect NCI-H2052 cell viability, in agreement with previous studies showing a resistance of this cell line to the infection with Ad5 derived oncolytic viruses (33).

**Figure 2:**
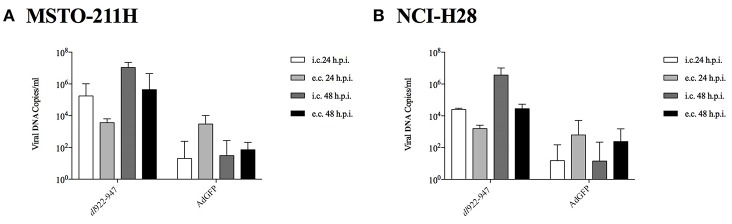
MSTO-211H **(A)** and NCI-H28 **(B)** cells were infected with *dl*922-947 and the non-replicating AdGFP. 24 and 48 hpi supernatant and adherent cells were collected separately to evaluate, respectively, extracellular (e.c.) released and intracellular (i.c) viral particles. Viral DNA was then extracted and used to quantify viral titer by Real-Time PCR. The data represent the mean of three different experiments.

NCI-H2052 infection with a non-replicating reporter adenovirus transducing GFP (AdGFP, 25 pfu/cell), followed by cytofluorimetric assessment of GFP emission, consistently showed that viral entry was not efficient in this cell line (data not shown) (34). Thus, we focused on MSTO-211H and NCI-H28 cell lines to study the mechanisms of action of *dl*922-947, which is able to efficiently amplify in both cell lines at 24 and 48 hpi. The efficacy of amplification is evaluated with respect to the non-replicating control AdGFP. The intracellular amplification is already enhanced at 24 hpi; however, the effect is more evident for both the intracellular and extracellular fractions at 48 hpi (Figure 2).

### *dl*922-947 Infection Affects Cell Cycle Progression

**Figure 3:**
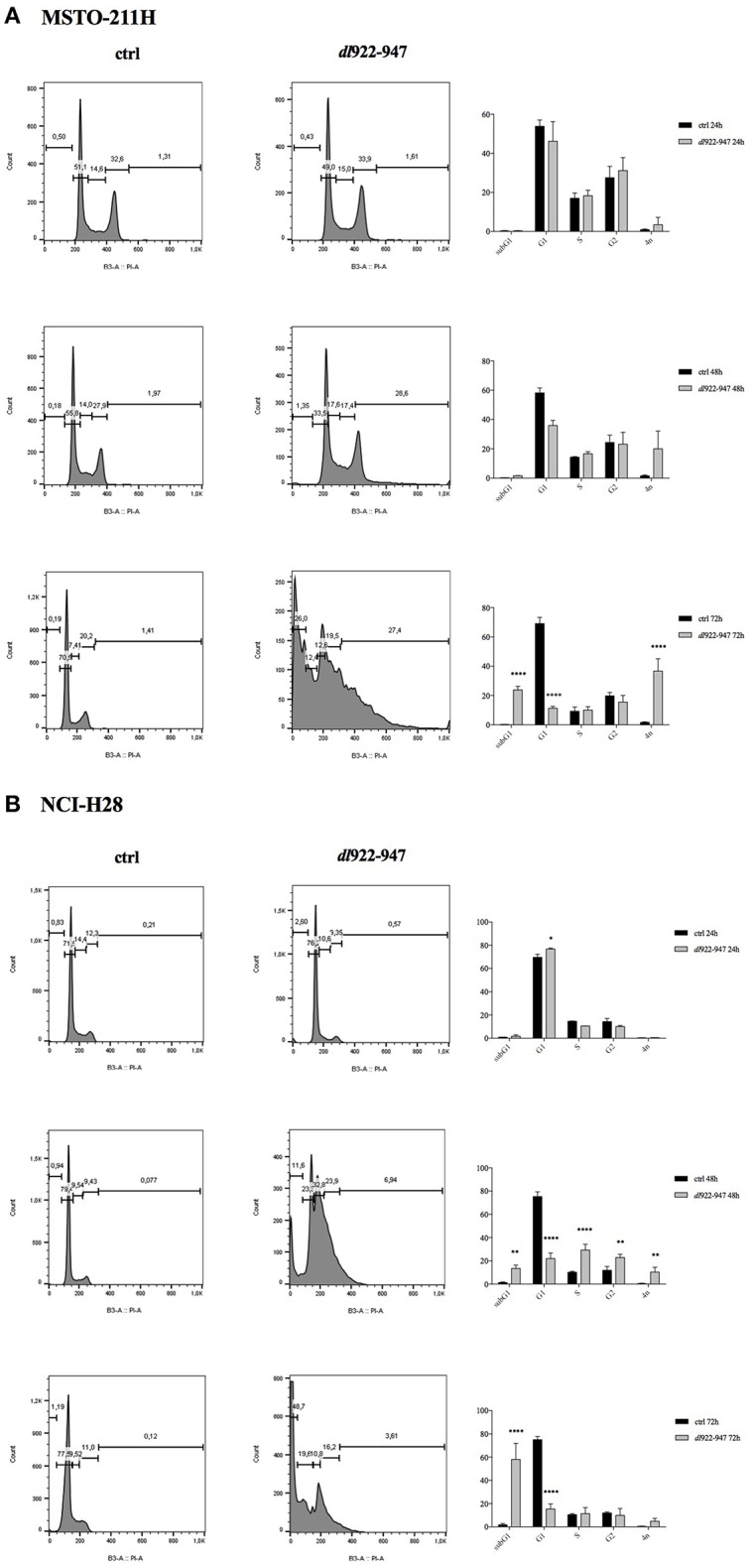
Effect of *dl*922-947 infection on cell cycle progression. MSTO-211H **(A)** and NCI-H28 **(B)** cells were infected with *dl*922-947 and compared with the uninfected controls by flow cytometry 24, 48, and 72 hpi. A representative cell cycle profile is shown on the left, the histograms on the right represent the means ± s.d. of at least three independent experiments. Statistical analysis was performed by two-way ANOVA with Sidak *post-hoc* test (*p < 0.01, **p < 0.005, ***p < 0.001, and ****p < 0.0001).

To evaluate the effect of *dl*922-947 on MPM cell cycle progression, cells were infected with *dl*922-947 and cultured for 24, 48, and 72 hpi. We used Adwt as control, and a representative cell cycle profile of the control showing no difference with respect to untreated cells is reported in Supplemental Figure 1 for the NCI-H28 cell line. The cell cycle profile of MSTO-211H was not significantly altered at 24 hpi, whereas at 48 hpi a decrease of the G1 phase was observed along with an increase of the sub G1 phase and >4N population, which were all maximal at 72 hpi (Figure 3A).

The cell cycle profile of NCI-H28 cells showed slight modifications at 24 hpi with an increase of the G1 phase. At 48 hpi the G1 phase decreased, with an increase of the subG1, S, G2 phases and >4N population. At 72 hpi the majority of cells accumulated within the subG1 phase (Figure 3B). Overall, although *dl*922-947 might act through different mechanisms in different MPM cell lines, as suggested by distinct effects on the cell cycle phases, it induced in both cell lines a significant increase of the subG1 phase, indicative of cell death, either by necrosis or apoptosis and an increase of the >4N population, indicative of mitotic defects. These effects are consistent with those obtained upon *dl*922-947 in other cancer cell lines (35, 36).

### ICD Induction by *dl*922-947 Infection: Intracellular ATP Content, Calreticulin, and HMGB1 Expression

Previous studies report that cell death induced by *dl*922-947 does not rely on classical apoptosis but rather shows features of regulated necrosis, although the underlying mechanisms still need to be clarified (37). Importantly, a paramount property of programmed necrosis is the ability to engage the host resident immune cells (38).

To assess whether *dl*922-947-induced cell death triggers the release of DAMPs and qualifies as ICD, suggesting that the virus can induce the recruitment of immune cells, we evaluated calreticulin exposure, intracellular ATP and HMGB1 production, the three DAMP hallmarks of ICD (16).

**Figure 4:**
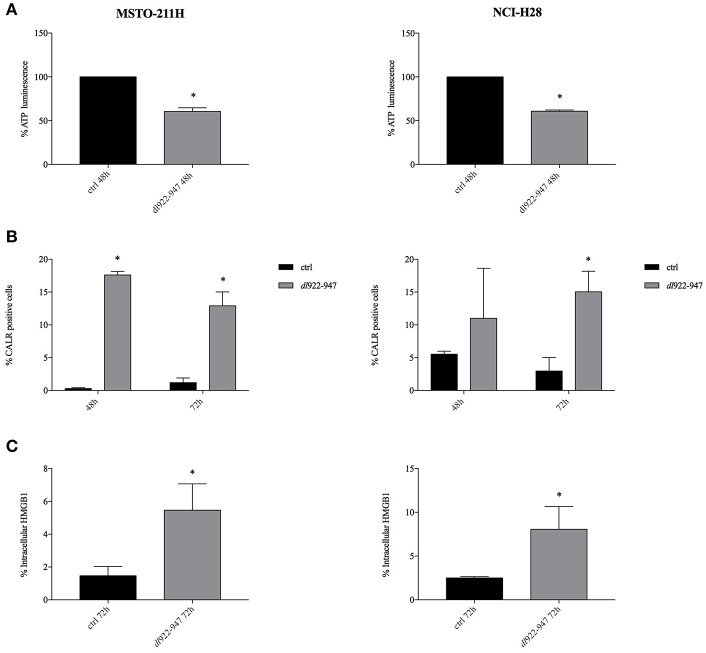
*dl*922-947 infection induces ICD in MPM cell lines. Intracellular ATP content **(A)**, calreticulin cell surface exposure **(B)**, and HMGB1 secretion **(C)** evaluated in MSTO-211H and NCI-H28 cells infected with *dl*922-947 at its IC50 values with respect to the control cells (black bar). In **(A)** the histograms report the luminescence percentage indicative of ATP content at 48 hpi. In **(B)** the histograms report the percentage of calreticulin positive MPM cells at 48 and 72 hpi (the mean fluorescence intensity, MFI is reported in Supplemental Table 1). In **(C)** the histograms report the percentage of HMGB1 positive cells at 72 hpi (MFI is reported in Supplemental Table 2). The histograms show the mean ± s.d. of three independent experiments. The statistical analysis was performed by *t*-test (*p < 0.05).

The exposure of the endoplasmic reticulum chaperone calreticulin on the surface of dying cells was observed in both MSTO-211H and NCI-H28 cells upon *dl922-947* infection along with reduced levels of intracellular ATP, implying its increased secretion (Figure 4) and Supplemental Table 1. HMGB1 expression was evaluated by flow cytometry after brefeldin A treatment to block extracellular secretion. An increase of intracellular HMGB1 in infected cells was observed at 72 hpi (Figure 4) and Supplemental Table 2 (data at 48 h not shown). The intracellular accumulation of HMGB1 after *dl*922-947 infection was confirmed by the lack of intracellular positivity in the absence of brefeldin A (data not shown). A representative histogram showing the positive control SAHA is reported in Supplemental Figure 2.

Overall these findings indicate that *dl922-*947 is able to induce ICD of MPM cells and therefore, through DAMP release, potentially able to trigger a cognate anticancer immune response.

### *dl*922-947 Infection Reduces the Production of the IL-8 and VEGF-A Pro-angiogenic Factors

**Figure 5:**
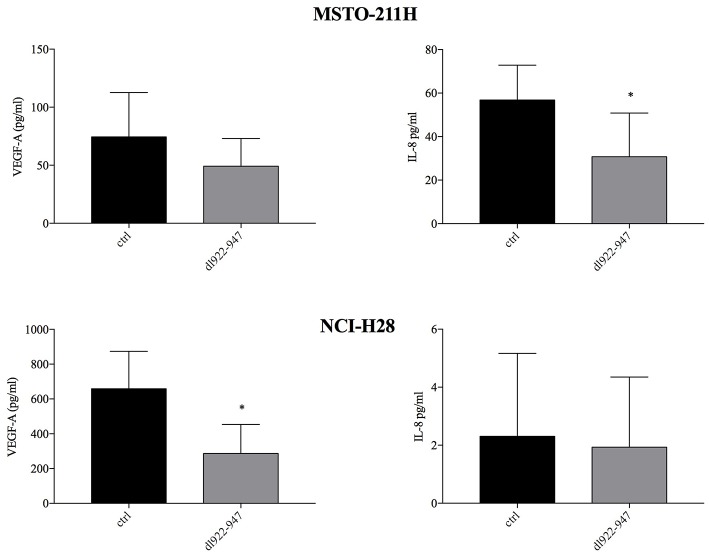
Effect of *dl*922-947 on VEGF-A and IL-8 production by MPM cells. MSTO-211H and NCI-H28 cells were infected with *dl*922-947 at the IC50. VEGF-A and IL-8 secretion was assessed by ELISA 48 hpi. The histograms represent the mean of six independent experiments ± s.d. The statistical analysis was performed by Mann-Whitney test (*p < 0.05).

We previously showed that *dl*922-947 exerts an antiangiogenic effect in anaplastic thyroid carcinoma cells (29). Therefore, we analyzed by ELISA whether *dl*922-947 infection was able to modulate the levels of IL-8 and VEGF-A pro-angiogenic factors, the latter correlating with poor survival in MM (39, 40). Indeed, *dl*922-947 infection proved effective in reducing significantly IL-8 production in MSTO-211H and VEGF-A production in NCI-H28 cells (Figure 5). Overall, the virus was effective in reducing these cytokines when they showed high basal levels.

### Treatment With *dl*922-947 Inhibits Tumor Growth *in vivo* in a Xenograft Model of MM

**Figure 6:**
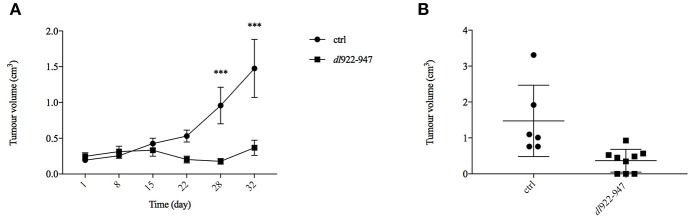
*dl*922-947 inhibits the growth of MPM xenografts. Mice were subjected to intra-tumoral injections with *dl*922-947 (4 × 10^6^ pfu) twice per week for a total of 5 weeks. **(A)** The days of treatment are reported; a significant difference (***p < 0.001) in tumor growth was observed at day 28. **(B)** Dot plot reporting the xenograft volumes at the final endpoint (32nd day of treatment). Three animals from the control group were sacrificed earlier because tumors were either too big or ulcerated, whereas 3 mice in the treated group showed complete tumor regression.

To extend further the preclinical characterization of *dl*922-947-based virotherapy against MM, we performed a pilot experiment in MM xenografts whereby athymic mice were inoculated subcutaneously with MSTO-211H cells. When the tumors became palpable, the mice were divided into two groups (of 9 animals each) and treated bi-weekly with *dl*922-947. Virotherapy proved extremely effective in counteracting tumor growth as early as after 3 weeks of treatment, achieving high statistically significant difference at the fourth and fifth (last) weeks of treatment (Figure 6A). The statistical analysis was performed by the Sidak *post-hoc* test.

**Figure 7:**
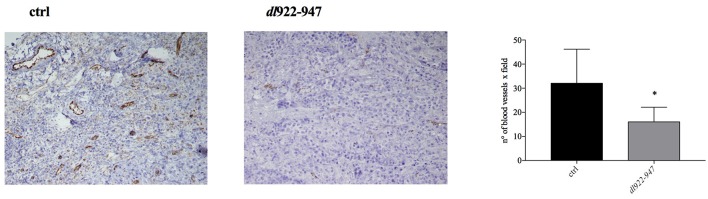
Histological analysis of CD31 expression. Tumor mass was collected to analyze CD31 endothelial marker presence. A representative image of control and *dl*922-947 treated mice is shown. The histogram reports the mean ± s.d. of TMD in tumor samples excised at day 32 treated as described above. TMD was determined as number of blood vessels x field. The statistical analysis was performed by Mann-Whitney *t*-test (*p < 0.05).

An animal treated with *dl*922-947 showed total tumor regression already after the first week of virotherapy, and other two mice had similar results during the course of treatment. Animals that had shown total tumor regression were not sacrificed at the end of the experiment and were observed for 3 additional months. During this period, no tumor re-growth was observed. The dot plot in Figure 6B reports the difference in xenograft volumes at the final endpoint (32nd day of treatment) between the two groups; however, the difference between the mean values might be higher considering that 3 animals from the control group were sacrificed earlier because the xenograft was either ulcerated or reached the maximal threshold allowed by our approved protocol. Additionally, immunohistochemical analysis showed a severe reduction of tumor microvessel density (TMD), evaluated as CD31 endothelial marker presence compared to controls (Figure 7).

### *dl*922-947 Synergizes With Cisplatin in MPM Cell Lines

**Figure 8:**
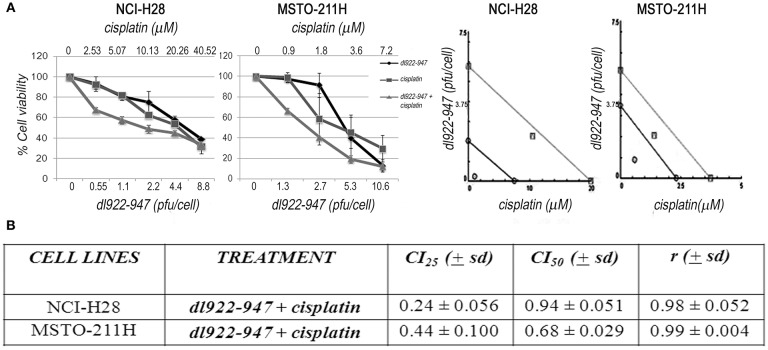
Synergistic effect of the *dl*922-947 and cisplatin combination. **(A)** MSTO-211H and NCI-H28 cell viability was analyzed by SRB assay 5 days after treatment with *dl*922-947, both alone and in combination with cisplatin. Left: Dose—response curves for *dl*922-947 alone, cisplatin alone and their combination. Results represent the means and standard deviations of two independent experiments, each conducted in triplicate, and are expressed as percentages of cell viability, calculated with respect to the control cells treated with DMSO alone. Right: Isobologram analysis to assess drug synergism. Isobolograms are derived from the mean values of the dose—response experiments reported in the left, through the CompuSyn software 1.0 at an effect level of 25 and 50%. Data points on the line indicate additivity; points below the line indicate synergy; points above the line indicate antagonism. **(B)** The table reports the means ± s.d. of the drug combination index (CI) at 25% (CI25) and 50% (CI50) of cell killing after 5 days of treatment and the *r*-values. CI values below 1 indicate drug synergism.

*dl*922-947 has been shown to interact synergistically with different drugs or various therapeutic approaches (41). Therefore, we also investigated by SRB the possible cytotoxic effects of *dl*922-947 in combination with cisplatin, which is the first-line treatment for MM, against which new possible therapeutic protocols should be confronted. By analyzing different schedules of treatment, we found that cisplatin treatment, added at 24 hpi, increased the cytotoxic effect of the OV both in NCI-H28 and MSTO-211H at day 5 (Figure 8), suggesting that virotherapy might be attempted along with the mainstay of treatment against MM.

## Discussion

MM is a very aggressive cancer, against which no curative modalities exist. Two main features contribute to the poor prognosis of MM: the disease is often diagnosed at an already advanced stage and is highly resistant to current standard therapeutic regimens and to the many experimental approaches attempted so far (42). Therefore, the development of new therapeutic strategies is urgently needed.

OVs represent a promising therapeutic tool considering that many clinical studies have demonstrated their efficacy and safety (10, 43). MM in particular is considered especially amenable to treatment with OVs and indeed many studies have evaluated the possible use of both replication competent and incompetent viruses against this disease (12).

Replication competent OVs, such as HSV, measles virus, NDV, reovirus and vaccinia viruses JX-594 and VV–IL-2 exert strong oncolytic activity and have been tested in MPM patients, providing encouraging results (12). ONCOS-102, an armed oncolytic adenovirus bearing a 24 bp deletion in the RB binding site of the *E1A* gene and engineered to express the granulocyte-macrophage colony-stimulating factor to enhance the immune-stimulatory effect, has been tested for MPM treatment. ONCOS-102 was able to induce ICD *in vitro* exhibiting anti-tumor activity in xenograft models (44). ONCOS-102 has been used in a phase I clinical study (45) with evidence of efficacy, safety and immunological activity (12).

The use of armed OVs transducing immune stimulatory genes has been proposed to boost a more robust immune response against the neoplastic cells. However, in the last few years, inhibitors of immunological checkpoints have been generated and their use in combination with OVs has been already tested in the clinic with encouraging results, making it necessary to assess in experimental models the treatment schedules. To this aim it is required to assess the direct effects of unarmed OV treatment on immune stimulation and on the TME, since the use of armed OVs, expressing immune stimulating genes, precludes the assessment of *per se* viral effects on innate and acquired immune response and on TME reshaping.

Therefore, we have assessed the effects of the unarmed *dl*922-947 oncolytic adenovirus in MPM. We found that *dl*922-947 efficiently amplifies and reduces cell viability of cell lines derived from epithelioid and biphasic MPMs, but not in NCI-H2052 representative of the sarcomatoid histotype. In this cell line, viral entry was not efficient, consistent with what has previously been reported in the literature and in agreement with the reduced expression of the coxsackie virus and adenovirus receptor (CAR), as frequently observed in advanced aggressive cancers (34). The replacement of CAR binding sequence of Ad5 with the CD46 binding sequence of Ad35 enhanced the infectivity, virus progeny production, and cytocidal effects (46), suggesting that a retargeted *dl*922-947 could be envisioned to increase viral entry and allow a wider use of this strategy against all MPM subtypes. Cell cycle analyses showed that *dl*922-947 subverts the host scheduled progression through the cell cycle phases and, although the underlying mechanisms seem to differ in MSTO-211H and NCI-H28, the virus induced in both cell lines a significant increase of the subG1 and >4N populations. The accumulation of such >4N population is due to the hijacked cell cycle regulation upon infection and is paralleled by the increase of the subG1 phase, which is associated with cell death. These data are in accordance with our previous findings dissecting *dl*922-947 mechanisms of action in cell lines derived from other cancer types (35, 36).

OVs trigger cell demise through various mechanisms engaging different host cell death machineries and depending on the virus or the host cells or by a combination of both. The mechanisms whereby *dl*922-947 induces cell death are only partially being revealed; recently, features of programmed necrosis different from canonical necroptosis have been described (37). However, beyond the underlying pathways, it is now recognized that a paramount mechanism of action of OVs is their ability to act as *in situ* cancer vaccines, releasing tumor antigens and activating a robust tumor-specific immune response, which is one of the major goals of current cancer therapies (47, 48).

The ability of OVs to induce ICD is key to stimulating the immune response in an “antigen agnostic” manner, that is, without previous knowledge of tumor neoantigens and therefore widely applicable (47, 49). Therefore, we evaluated whether *dl*922-947 might activate ICD in MPM cells.

We show that *dl*922-947 was able to modulate the three hallmarks of ICD in MPM cells affecting the release of ATP (“find me” signal), which enhances the recruitment and activation of dendritic cells, calreticulin exposure (“eat me” signal) which promotes phagocytosis, and the expression of HMGB1 whose release promotes dendritic cell maturation (49).

Interestingly, HMGB1, released by dying cells following exposure to asbestos fibers, has a fundamental role in MM pathogenesis supporting the chronic inflammation that fosters tumor development and immune suppression (50–52). However, while chronic HMGB1 release is detrimental, an acute and sudden release following OV infection triggers tumor-specific immunity with beneficial effects (47).

In both cell lines a similar decrease of intracellular ATP was observed. Calreticulin exposure was reduced in MSTO-211H cells at both 48 h and 72 h, which is consistent with the increase of subG1 phase of the cell cycle observed at 72 h, since calreticulin is a pre-mortem signal. NCI-H28 cells express a slightly higher basal level of calreticulin and although we observed an increase at 48 h, statistical significance was reached only at 72 h, despite the increase of subG1 phase being detected also at 48 h. HMGB1, a signal secreted by dying cells, was increased by the virus in both cell lines at 72 h, consistently with the increase of subG1 phase of the cell cycle at 72 h.

We previously showed in an ATC experimental model that *dl*922-947 downregulates the expression of IL-8 interfering with NF-κB binding on its promoter, thereby impairing tumor angiogenesis (29). So, we assessed whether *dl*922-947 might modulate angiogenic signals also in MPM cells. The virus indeed proved able to decrease the levels of IL-8 or the pro-angiogenic factor VEGF-A when they were expressed at high basal levels in MSTO-211H and NCI-H28 cells. In particular, in MSTO-211H cells we observed a significant reduction of IL-8 secretion, whereas the decrease of VEGF did not reach statistical significance. In NCI-H28 cells, IL-8 was not secreted or the secretion was too low to detect any effect of the virus, instead this cell line produces high amounts of VEGF that in this case is significantly reduced by virus treatment. IL-8 was found highly expressed in pleural fluids from MM patients (53) and its direct inhibition was shown able to reduce MM growth in mice (54). High VEGF-A serum level correlates with poor survival in MM and has been proposed as a biomarker to identify who among asbestos-exposed individuals is more prone to developing MM (39, 40). These data support an anti-angiogenic activity of *dl*922-947 in MM cells and, considering the pivotal role that both IL-8 and VEGF play in MM, the use of *dl*922-947 could have yet another advantage to tackle the disease.

To validate our *in vitro* preclinical data we settled an *in vivo* murine model to address the anti-tumor efficacy of *dl*922-947 in tumor-bearing mice. In this xenograft model, *dl*922-947 efficiently reduced tumor volume and increased survival. Complete tumor regression was achieved in 30% of the animals. Moreover, the remaining animals bore tumors of a very small volume, and it is likely that prolonged treatment would have allowed the tumors to decline in a greater number of animals. Immunohistochemical analysis showed a severe reduction of TMD compared to controls, further confirming the antiangiogenic effects exerted by *dl*922-947. Combination strategies with currently used antineoplastic drugs (25), ionizing radiation (36), and anti-angiogenic drugs (28) can strengthen the potential anti-tumor effect of OVs. Therefore, we tested whether *dl*922-947 infection could work in combination with cisplatin, which represents the mainstay of treatment against MM (55). We found that cisplatin treatment following infection with *dl*922-947 increased viral cytotoxicity showing synergism as assessed upon testing a wide range of doses. The efficacy of this combined treatment has already been assessed using conditionally replicating viruses or retargeted viruses (56, 57). The armed OV ONCOS-102 has also been used in combination with both cisplatin and pemetrexed in a xenograft model of human MM showing a synergistic anti-tumor effect (44). Overall, our data confirm the efficacy of the combination in MPM cells and demonstrate that the unarmed *dl*922-947 virus potentiates the effects of cisplatin alone. These findings thereby set the basis for the potential use of these agents in combination, which could reduce possible side effects.

In conclusion, our data show that the unarmed OV *dl*922-947 is effective alone against MPM cells in inducing the activation of ICD, reduces the secretion of angiogenic factors and stimulates an innate immune response. Therefore, *dl*922-947 could be a feasible strategy against MM in combination with other therapeutic agents/strategies. The potential use of *dl*922-947 to potentiate ICD and anti-tumor immunity working in combination with other ICD inducers or other immunotherapy strategies deserves further investigation.

## Data Availability

All datasets generated for this study are included in the manuscript and/or the Supplementary Files.

## Ethics Statement

All animal experiments were conducted in compliance with the Italian current regulations for the welfare of animals used in studies of experimental neoplasia. The study was approved by our institutional committee (OBPA) on animal care and by the Ministero della Salute (authorization n. 605/2017-PR).

## Author Contributions

SD and CI carried out most of the experiments and analyzed the data. CP generated the OV and contributed to data analysis. IF and PI performed the drug combination experiments and contributed to data analysis. RI collaborated on the *in vivo* experiment. VG and GB performed and evaluated xenograft immunohistochemical analyses. AG and PF supervised the work and provided critical feedback. AM performed the ICD characterization and drafted the manuscript. GP and FP conceived and planned the experiments, interpreted the data and wrote the manuscript. All authors read and approved the final manuscript.

### Conflict of Interest Statement

The authors declare that the research was conducted in the absence of any commercial or financial relationships that could be construed as a potential conflict of interest. The handling Editor declared a past co-authorship with the authors AM and GP.

## Abbreviations

Ads: adenoviruses
ATC: anaplastic thyroid carcinoma
CAR: coxsackie virus and adenovirus receptor
CI: combination index
DAMP: damage-associated molecular pattern molecules
HDAC: histone deacetylase
HPI: hours post-infection
HMGB1: high mobility group box protein 1
IC50: half maximal inhibitory concentration
ICD: immunogenic cell death
IFN: interferons
MM: malignant mesothelioma
MPM: malignant pleural mesothelioma
PAMP: pathogen-associated molecular pattern
OV: oncolytic virus
PI: propidium iodide
RB: retinoblastoma
S.D: standard deviation
SEM: standard error of the mean
SRB: sulforhodamine B
TAA: tumor-associated antigen
TAM: tumor-associated macrophage
TMD: tumor microvessel density
TNF: tumor necrosis factor
TME: tumor microenvironment
VP: viral particles
WHO: World Health Organization.
