# Supplementary material for: The Oncolytic Virus dl922-947 Triggers Immunogenic Cell Death in Mesothelioma and Reduces Xenograft Growth
Source: Front Oncol. 2019 Jul 12;9:564. doi: 10.3389/fonc.2019.00564 (PMC6639422; doi:10.3389/fonc.2019.00564)
Supplement: Supplementary file 1 [file Data_Sheet_1.docx]

**Supplementary Tables**

Table 1. MFI values of calreticulin expression in MSTO-211H and NCI-H28 cells in the presence and in the absence of dl922-947.

|  | MSTO-211H (48h) | MSTO-211H (72h) | NCI-H28 (48h) | NCI-H28 (72h) |
| --- | --- | --- | --- | --- |
| Ctr | 412±7,1 | 1155±262,3 | 4237±294,8 | 2028±57,9 |
| dl-922-947 | 488±40,3 | 1635±39,5 | 9543,5±477,2 | 4693±84,1 |

Legend of Table1

In the table we reported the values of MFI related to the calreticulin expression in both cell lines in the presence and in the absence of dl922-947. The values are reported as mean of three independent experiments.

Table 2. MFI values of intracellular HMGB1 accumulation in MSTO-211H and NCI-H28 cells in the presence and in the absence of dl922-947.

|  | MSTO-211H (72h) | NCI-H28 (72h) |
| --- | --- | --- |
| Ctr | 614±6,3 | 582±9,8 |
| dl-922-947 | 714±93,3 | 716±65,7 |

Legend of Table 2

In the table we reported the values of MFI related to the HMGB1 accumulation in both cell lines in the presence and in the absence of dl922-947. The values are reported as mean of three independent experiments.

**Supplementary Figure Legends**

Legend of Fig .1

A representative flow cytometry profile of control (untreated) cells, cells infected with Adwt (used at IC50 of dl922-947), and dl922-947 (IC50) is reported in particular for NCI-H28 cells at 24hpi, 48hpi and 72hpi.

Legend of Fig. 2

1. We report a representative histogram showing the reduction of intracellular ATP of the virus with respect to a positive control, SAHA (5µM) in both MSTO-211H and NCI-H28 cells at 48 hpi. B) flow cytometry profile of calreticulin exposure in both MSTO-211H and NCI-H28 cells at 48 hpi and 72 hpi is reported with respect to SAHA (5µM). The percent of parental cells is reported in each histogram. C) A representative flow cytometry profile of MSTO-211H and NCI-H28 cells treated with dl-922-947 (IC50) and SAHA (5µM) is reported with the indication of the percent of parental cells that are positive for HMGB1 at 72 hpi.
