# Supplementary figures and images for: The Oncolytic Virus dl922-947 Triggers Immunogenic Cell Death in Mesothelioma and Reduces Xenograft Growth
Source: Front Oncol. 2019 Jul 12;9:564. doi: 10.3389/fonc.2019.00564 (PMC6639422; doi:10.3389/fonc.2019.00564)

Figure 1

24 h

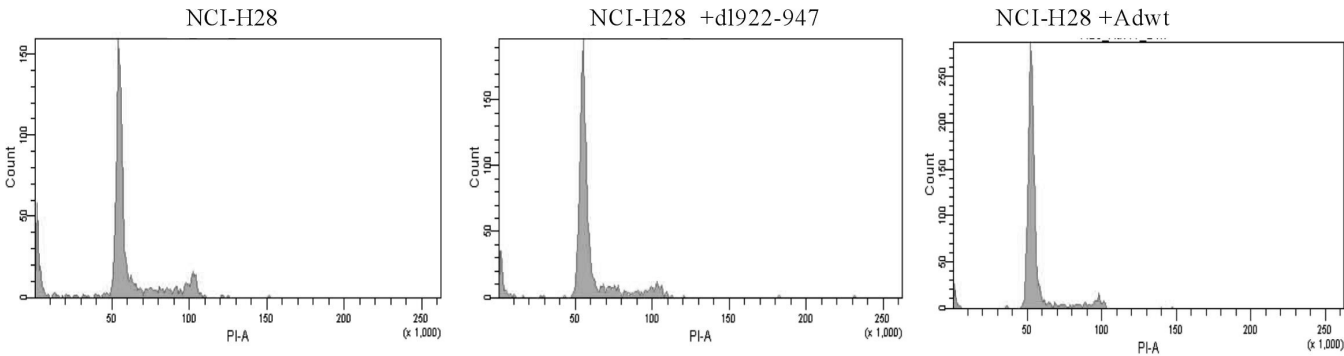

48 h

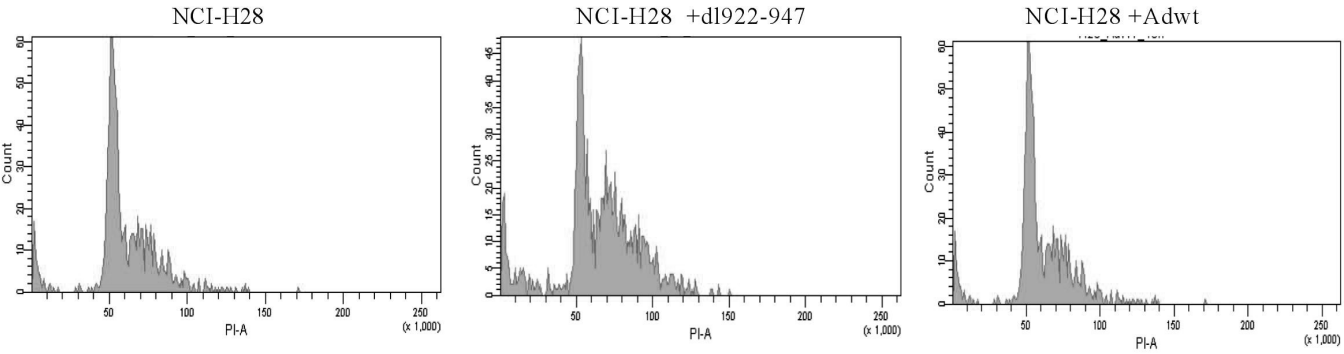

72 h

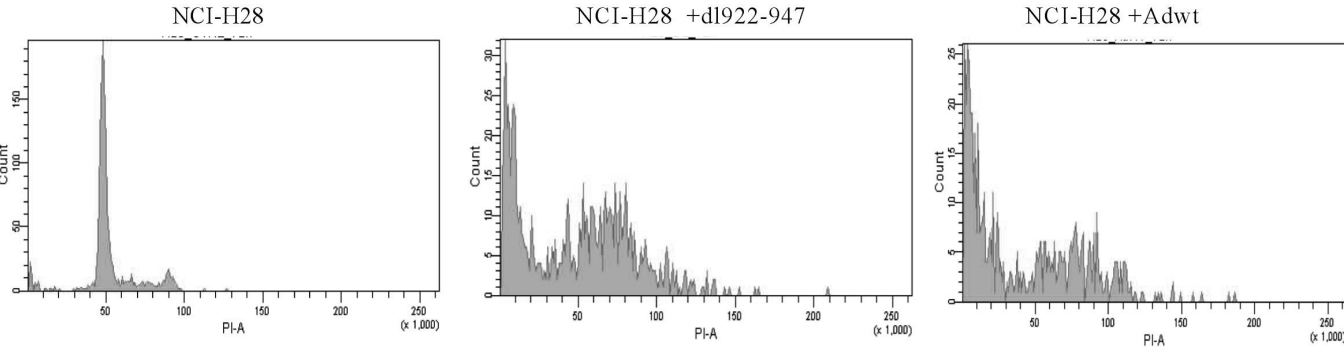

Figure 2

A

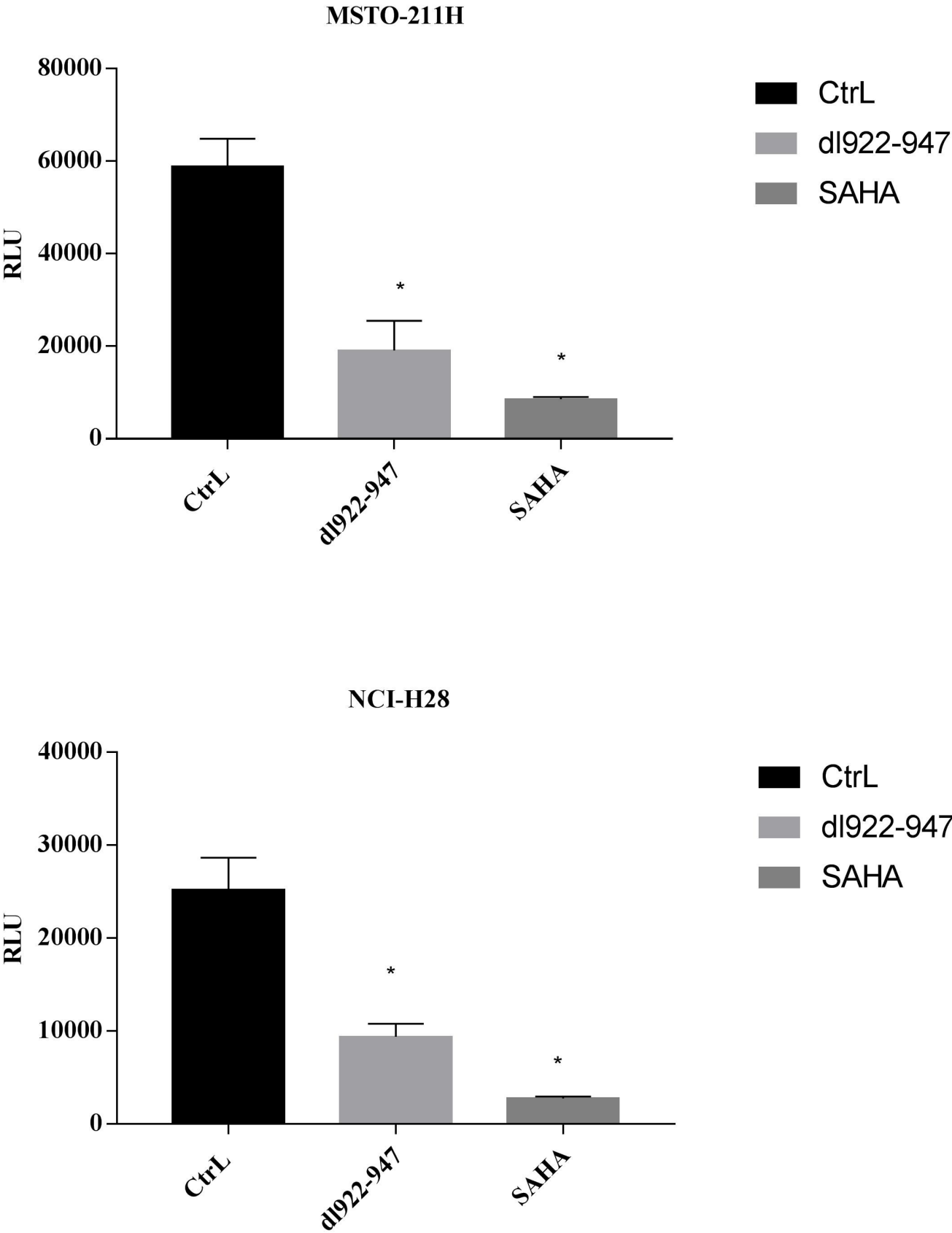

**B**

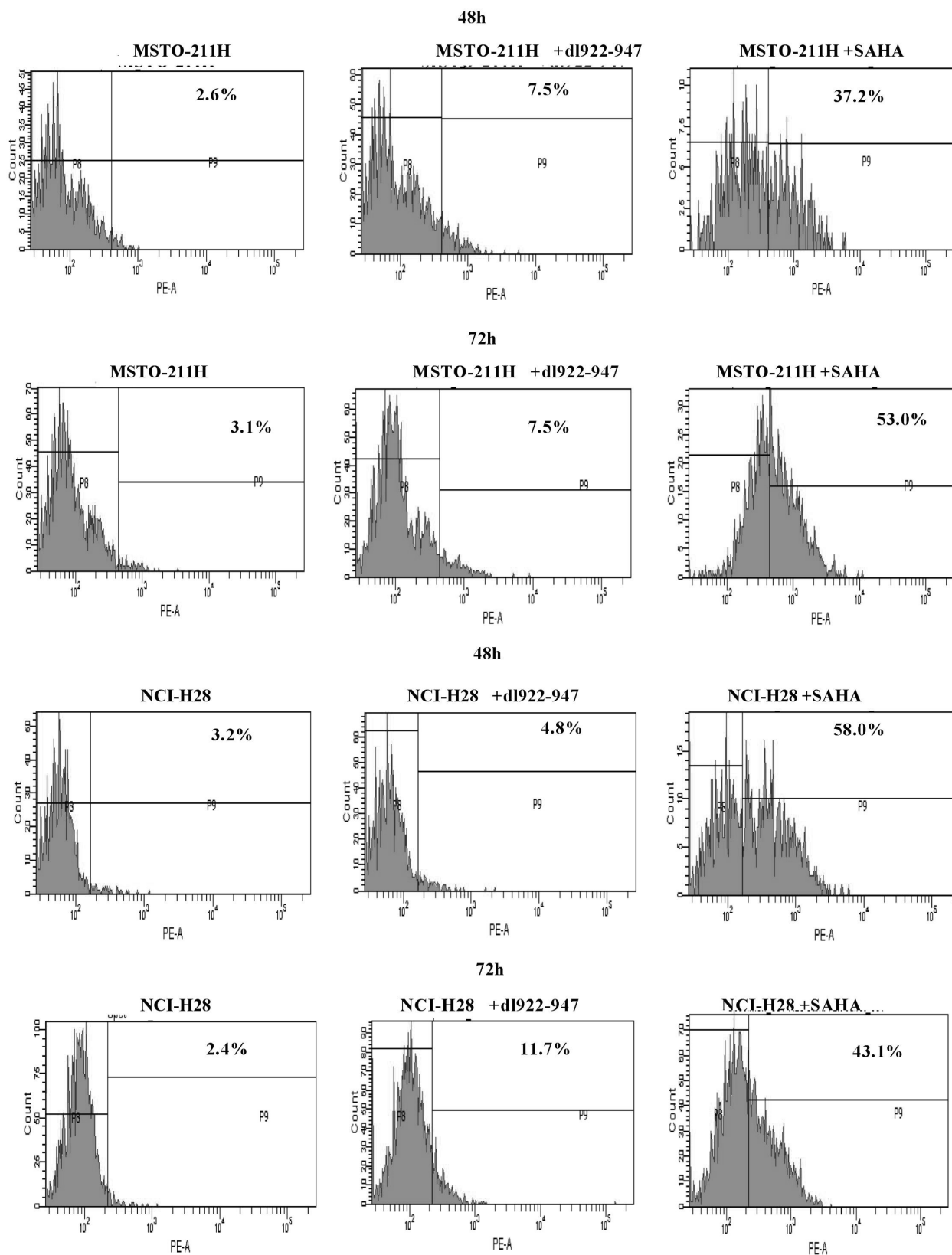

C

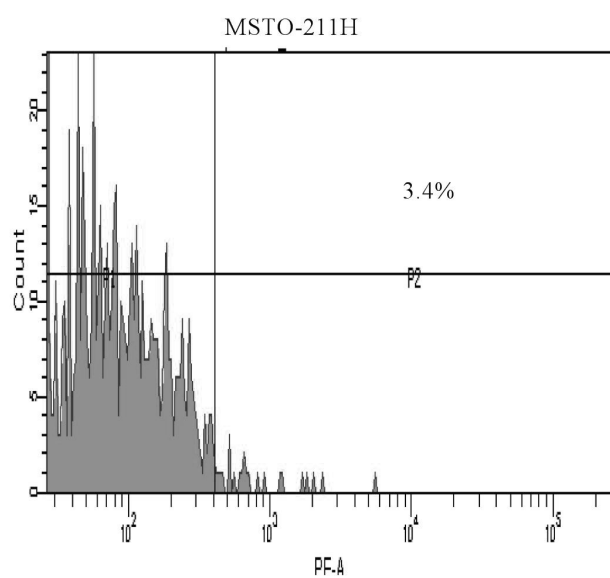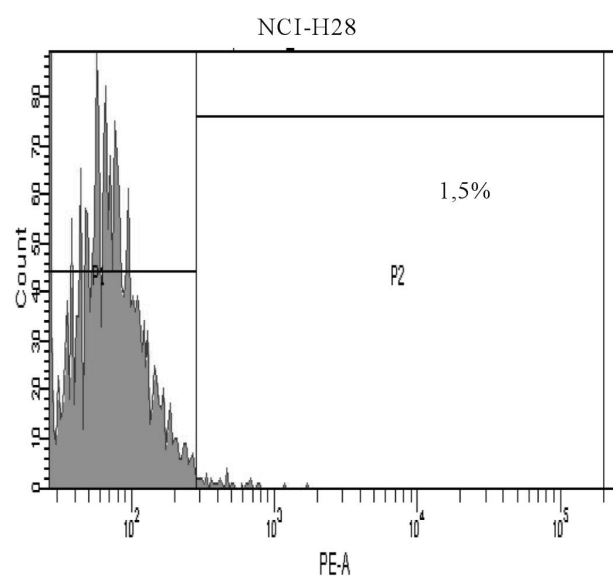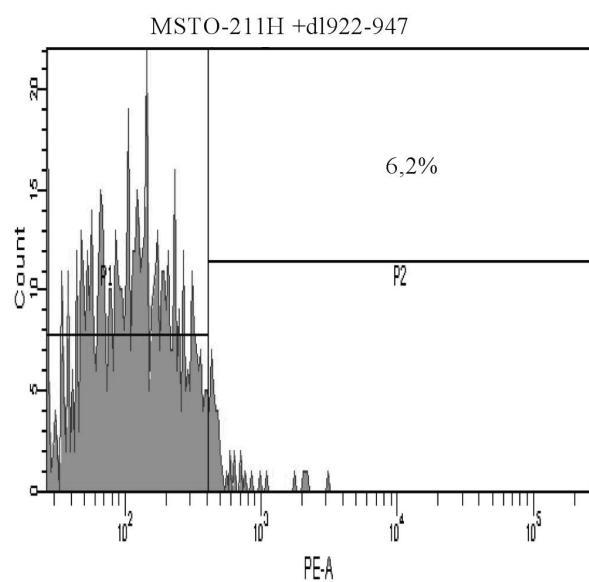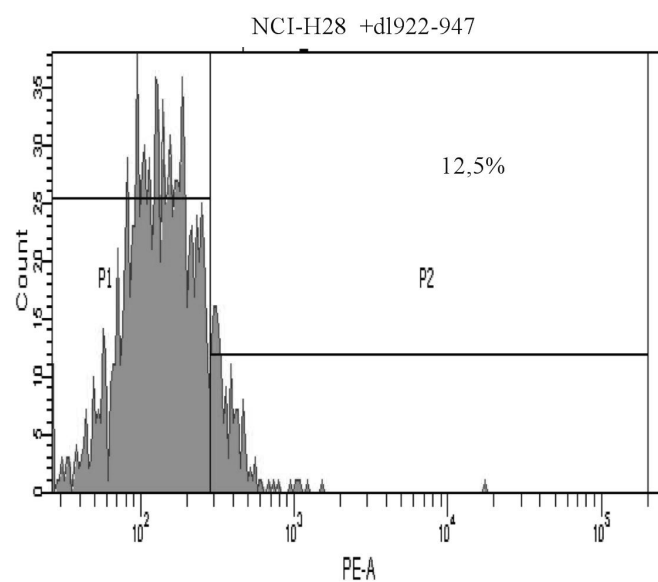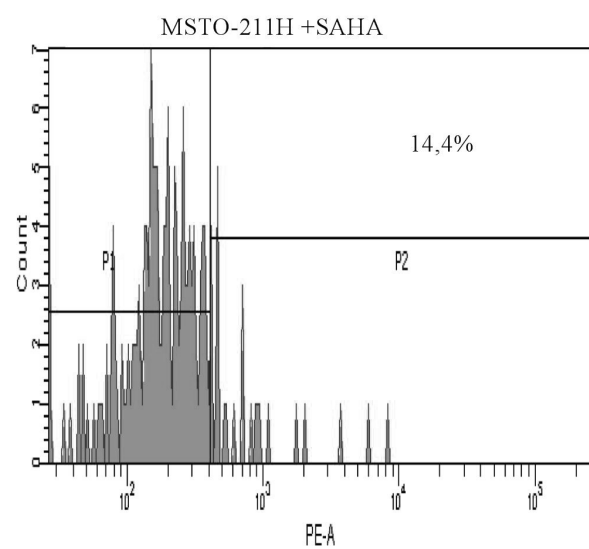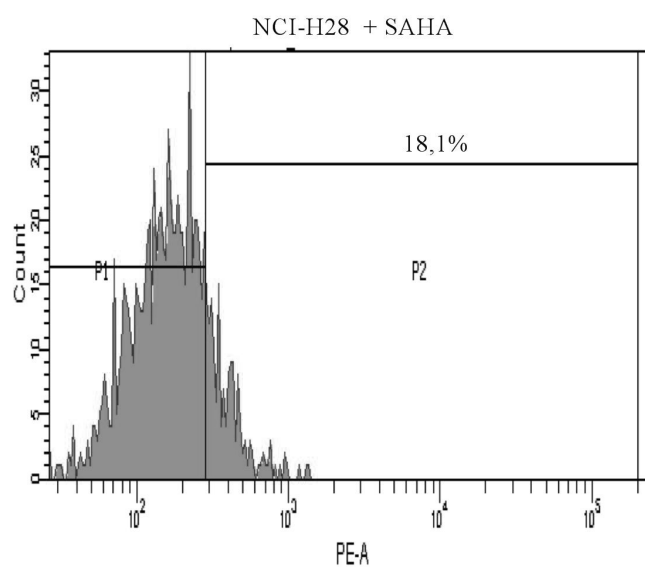

Supplement: Supplementary file 2 [file Data_Sheet_2.pdf]
